# The impacts of working from home on individual health and well-being

**DOI:** 10.1007/s10198-023-01620-8

**Published:** 2023-08-30

**Authors:** Manuel Denzer, Philipp Grunau

**Affiliations:** 1 Gutenberg School of Management and Economics, Johannes Gutenberg-University Mainz, Jakob-Welder-Weg 4, 55128 Mainz, Germany; 2 Institute for Employment Research, Regensburger Straße 100, 90478 Nürnberg, Germany

**Keywords:** Working from home, Health, Well-being, C26, I10, I31, O33

## Abstract

Using a novel German linked employer–employee dataset, we provide unique evidence about the consequences of working from home (WfH) on individual health and well-being. During the recent pandemic, this locational flexibility measure has been used extensively to promote health by hampering the spread of the virus and to secure jobs. However, its direct theoretical ambiguous effects on health and well-being as characterized by different potential channels have barely been empirically investigated to date despite WfH’s increasing popularity in the years before the pandemic. To address concerns about selection into WfH in our dataset that is unaffected by the COVID-19 shock, our analysis relies on an identification strategy ruling out confounding effects by time-invariant unobservable variables. Moreover, we explain the remaining (intertemporal) variation in the individual WfH status by means of an instrumental variable strategy using variation in equipment with mobile devices among establishments. We find that subjective measures of individual health are partly affected by WfH, whereas no corresponding effect is present for an objective measure of individual health. In terms of individual well-being, we find that WfH leads to considerable improvement. By addressing the potential heterogeneity in our effect of interest, we find that men and middle-aged individuals particularly benefit from WfH.

## Introduction

**Fig. 1:**
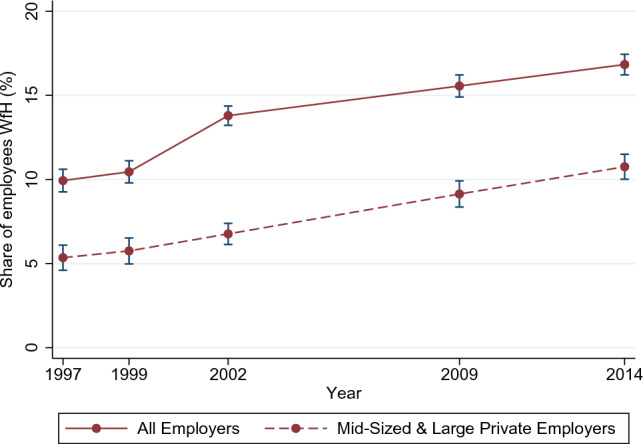
Trends in WfH. From German Socio-Economic Panel (SOEP). The group of *mid-sized and large private employers* consists of all employees working for non-public employers with 20 or more employees. Individuals are considered WfH if they use this option at least once a month

Since the outbreak of COVID-19 and the resulting physical distancing and confinement measures, the way in which jobs are performed has changed considerably among different occupations, sectors, and countries. Many employees were forced to move their workplaces to their homes from one day to the next, often even full-time. Even two years after the outbreak of COVID-19, physical distancing measures are still ongoing, and the level of individuals working from home (WfH) is quite high [1–4]. However, taking a closer look reveals that even with this sudden increase in WfH during 2020 for West European and North American countries, WfH has already become steadily more popular over recent years and decades. Previous studies report considerable increases in corresponding indicators for both US as well as European data [5–8]. Figure 1 underlines this trend in WfH by exemplarily plotting it for employees in Germany using a representative sample of its population. It shows that in the years until 2014, which are the years prior to those considered in this analysis, the share of German employees WfH increased substantially and steadily, from 10% in 1997 to about 17% in 2014. Moreover, Fig. 1 shows that this trend also holds for employees working for mid-size and large private employers, who are the focus of this investigation. For this subgroup, the trend seems to be almost perfectly linear.^1^

In principle, the costs of working remotely have decreased considerably in recent years. Faster broadband connections and progress in information and communications technology (ICT) made it easier to regularly work without being present in the office [9, 10]. As a result of the ongoing digitization, many occupations also experienced a shift in tasks composition, which allows workers to work remotely. In addition, the shift toward a higher demand for services instead of goods for Western European and North American countries has contributed to the increased possibility of WfH [11]. From a company’s perspective, increasing rents for offices in metropolitan areas as well as increasing travel costs may have highlighted the cost-reducing function of WfH as a measure of becoming more profitable [12]. Another competitive advantage can come from being more attractive as an employer if skilled workers demand that they be allowed to perform part of their work from home. Last but not least, worker shortages in general have led employers to rethink their work organization to enable parents, particularly mothers, to participate in the labor force while taking care of their children [11].

While measures such as physical distancing have been shown to be effective in terms of COVID-19 containment (see Chu et al. [13] and Flaxman et al. [14] for some meta-analyses and particularly Weber [15] and Kosfeld et al. [16] for the evaluation of measures taken by the German government) and thus to protect and promote individual health during the pandemic, less is known about the direct impacts of WfH as a major instrument of physical distancing for ensuring the current functioning of economies. Although this paper examines the impact of WfH on health using pre-COVID-19 data, our findings have important implications for the tremendous changes to working environments induced by the current pandemic. Direct effects on individual health have rarely been investigated despite the popularity of this work practice as described above. The same is true for measures that do not merely cover physical aspects of health but also mental and social aspects. The previous literature has primarily focused on the investigation of WfH effects on more traditional outcomes, such as the labor supply and wages, as well as job or life satisfaction.^2^

From a theoretical perspective, the effects of WfH on individual health and well-being are ambiguous. In general, WfH is expected to increase flexibility, which, in turn, might affect labor as well as health outcomes. On the one hand, WfH might have a positive impact on individual health and well-being through the following channels. First and most intuitive, WfH eliminates commuting and the corresponding stress. Second and related, in the case of WfH, the time that does not have to be spent commuting can be used for regeneration or physical activity, such as exercising, promoting total health. Moreover, commuting might be characterized by adverse health effects such as exposure to air pollution due to traffic jams when riding by cars. Third, WfH can help workers to reschedule constraints as well as to reconcile family and work life, particularly in the presence of young children. Consequently, it might improve the individual’s work–life balance. Fourth, WfH might allow patients to recover from surgeries or illnesses smoothly at home instead of providing full working hours. Fifth, a reduction in social contacts due to WfH might lead to being less exposed to infectious diseases such as, most recently, the COVID-19 virus, but in principle other regular diseases such as classical influenza. Finally, WfH might ease the caring challenges associated with individual health issues or disabilities, especially in the case of severe handicaps.

On the other hand, individual health and well-being might be negatively affected by WfH. First, WfH can be harmful by increasing the individual stress level. Working and living in different places enable us to separate both crucial parts of daily life and to tune out issues and problems related to one part while being active in the other. This separation vanishes during high-intensity WfH and might result in increased stress when employees are trying to cope with both issues simultaneously. Second, WfH could also lead to excessive working beyond the usual office hours, which in the long run might have adverse effects when experienced on a substantial level. Third and related, WfH could release the pressure to be permanently reachable also on weekends. Due to corresponding concerns, some prominent companies have decided to shut down their mail servers on weekends to protect their employees’ health.^3^ Fourth, WfH might amplify isolation and loneliness. Although video conferencing via the internet has become a widely accepted and important tool for interpersonal interaction, real social interaction continues to be an elementary need of humans. Fifth, WfH can be characterized by having bad posture and inappropriate work surfaces, when the employers are not legally obliged to take care of these issues. Sixth and last, the positive aspect of WfH in the case of minor sicknesses might be beneficial in the short run but might have negative health consequences in the long run when employees do not take enough downtime.

In this paper, we empirically investigate the effects of WfH on individual health in terms of a subjective overall health measure, the number of days being sick, and well-being as proxy for mental health. Given the outlined potential opposing channels, we primarily focus on the overall effect of WfH. By considering potential impacts on well-being, we do not narrow our analysis to physical health, as outlined above. The differentiation between subjective and objective health outcomes will reveal any related perception effects. The analysis rests on a representative and novel data product for personnel economic research in Germany of the Institute of Employment Research Nuremberg (IAB) which is additionally enriched by administrative data. This approach allows us to consider the personal characteristics influencing individual health and well-being as well as the WfH decision and employer characteristics, which matter because employers need to allow their employees WfH from a legal perspective and to provide the corresponding technical possibilities. The time frame of our analysis (2015–2019) maps to a period during which WfH exhibits an ongoing increase in popularity but is not affected by the COVID-19 shock, which would question the external validity of the results for times when the COVID-19 pandemic has come to an end.^4^

To the best of our knowledge, the impacts of WfH on individual health outcomes have barely been investigated thus far. Hence, as the primary and first contribution, our analysis helps to complement the picture of the consequences of WfH on key individual outcomes. More precisely, this study supports policy makers and companies by yielding insights on WfH effects on outcomes that are not directly related to labor issues. This aspect is important since due to the COVID-19 shock and corresponding employer reactions, such as improving employees’ technical equipment, many companies, especially larger ones, are already planning a permanent extension of WfH for their staff in a post-pandemic era. Moreover, given the relatively long and ongoing period of confinements, habituation effects from the perspective of employees expressing ongoing demand for locational flexibility seem to be likely.

Second, the identification strategy of our study accounts for concerns of endogeneity in the individual’s decision of WfH. There are several reasons to believe that the decision of WfH cannot be considered as randomly distributed among individuals but is correlated with (unobserved) individual heterogeneity. To address this issue, our study exploits longitudinal data. This allows us to control for time-invariant heterogeneity at different levels, such as the establishment or the occupational level, in addition to controlling for a large set of time-varying individual socio-demographic and employment characteristics. To ensure that our results are not driven by any relationship between WfH and time-varying individual unobserved heterogeneity, our identification strategy additionally comprises an instrumental variable (IV) approach.

Third, given the variety of potential channels described above, we address heterogeneity in the effects of WfH on individual health outcomes and well-being. We particularly focus on the heterogeneous effects of WfH by gender, parenthood, age, and commuting behavior. This focus will yield more detailed insights into the potential individual benefits and drawbacks of WfH. Moreover, it links our analysis to the strand of literature investigating the relationship between gender or parenthood and the place of work [18] as well as the relationship between commuting and individual health [19].

Our study shows that WfH positively affects the measures of subjective perceived health. Regarding the measure of objective health, we find negative but insignificant point estimates of WfH on the number of days being sick, complementing the picture of non-negative effects of WfH on individual health. Moreover, WfH contributes to an improvement in individual well-being. Those individuals who are WfH exhibit an average increase in a corresponding index’s standard deviation of 0.64 in contrast to those who are not WfH or are not able to do so.

The remainder of this paper is structured as follows. In sect. “Literature”, we review the previous literature on the relationship between WfH and individual health and well-being. Section “Data and descriptives” describes the data used in our analysis and presents some descriptive statistics. We explain our empirical strategy in section “Empirical approach”. Section “Results” presents our results, while the last section, section “Conclusion”, concludes and provides ideas for further research.

## Literature

There is a large body of literature on the determinants and consequences of WfH, or more generally flexible work arrangements, in different disciplines, such as sociology, psychology, and economics. In contrast to studies from other disciplines (e.g., Chandola et al. [20], Li and Wang [21]) that are not the focus of this literature review, previous empirical economic studies on the consequences of WfH have primarily focused on labor market-related outcomes at the individual level. Foremost, the impact on wages and earnings is investigated in a couple of studies, such as Gariety and Shaffer [22], Glass and Noonan [23], Pigini and Staffolani [24], Arntz et al. [25], and Pabilonia and Vernon [26]. Relatedly, a large group of existing investigations focuses on the effects of WfH on working time, such as actual working time or overtime [11, 25]. In a similar vein, some studies concentrate on the relationship between WfH and individual work effort [27]. Other groups of studies examine the impact of WfH on job satisfaction [12, 25, 28–31] and life satisfaction [25, 32]. Finally, studies by Dutcher [33] and Bloom et al. [12] address the consequences of WfH for individual working performance as well as productivity and the corresponding consequences on company profitability. Most of those studies emphasize the possible endogeneity arising from non-random selection into WfH, but only a handful is able to control for it sufficiently.

Another strand of previous related economic literature investigates the determinants of absenteeism as an outcome of bad health or well-being. Most corresponding studies ignore the location of work as an important determinant for explaining absence from work. Among others, the impact of labor market tightness [34], labor market composition expressed by the unemployment rate [35], workplace characteristics, also reflecting those of colleagues [36], or statutory sick pay levels as well as other aspects of sick pay insurance [37–39] on measures of absenteeism has been studied.

The previous literature connecting both strands, i.e., the literature on how WfH affects individual health and well-being, as most often measured by absence rates, is rather limited. Only a handful of studies explicitly addresses the relationship between WfH and individual health. Some of them, in particular those from disciplines other than economics, only present descriptive and unrepresentative evidence leading to data-based associations but not to causal effect assignment. Moreover, others are based on reduced form analyses investigating the effect of having the possibility of WfH, for instance as technically enabled or allowed at the company or establishment level, instead of actual individual WfH behavior to circumvent any selection bias as described above. Therefore, these research designs for estimating intention-to-treat (ITT) effects come at the cost of providing diluted estimates of the true WfH effects.

Using establishment-level data from the United Kingdom (UK), Gray [40] analyzes the relationship between different family-friendly policies leading to an individual’s increased flexibility and different establishment performance measures such as absence rates. According to her findings, other family-friendly policies, such as providing a workplace nursery, have an impact on absenteeism, whereas local flexibility represented by the opportunity of WfH has no impact. Using the same dataset, Dex et al. [41] arrive at the same result. In a similar vein, Heywood and Miller [42] also use British establishment level data to examine the relationship between schedule flexibility and reported absence. In contrast to Gray and Dex et al., they find that establishment-level policies allowing WfH leads to reduced worker absence. However, they emphasize that their estimates have to be considered with caution due to endogeneity concerns regarding schedule flexibility at the establishment level.

To the best of our knowledge, the study by Dionne and Dostie [43] is the first to analyze the relationship between health and WfH systematically at the individual level. Using Canadian linked employer–employee data, the authors examine the effect of seven different workplace arrangements on the individual number of days being absent from work. Among others, they estimate the effect of having a work-at-home option and find that it is associated with reduced absence. Given their specific count data model, Dionne and Dostie explicitly control for individual and workplace heterogeneity to reduce any threats of identification by confounders.

Possenriede et al. [44] use cross-sectional individual data from the Dutch Public Sector Employee Survey 2004 to estimate the effect of being able WfH from time to time on both the individual absence frequency and the absence duration as measured in days. Using negative binomial regression models to explain their count outcomes characterized by overdispersion, they find that having the possibility of WfH is negatively associated with sickness-related absence frequency but not with sickness absence duration. However, as noted by Possenriede et al., their estimates cannot be seen as causal given that they are not able to control for individual-, job- or firm-related heterogeneity.

The above-mentioned study by Bloom et al. [12] examines the impacts of WfH on different outcomes by making use of a randomized control trial (RCT) in a large Chinese company. The study’s primary focus is on the impact of WfH on working productivity. By examining effect mechanisms, Bloom et al. provide evidence that the identified increase in productivity is partly due to a decrease in the workers’ number of (paid) sick days. According to their post-experimental survey, workers most often use the possibility of WfH when they are too sick to come to the office but can perform some of their job tasks and duties when working remotely, suggesting a negative effect of WfH on the number of sick days.

Kröll and Nüesch [31] ﻿use data from a representative panel survey of German individuals to analyze the relationship between several flexible work practices and job or leisure satisfaction as well as turnover intention. By considering WfH as one of three different flexible work practices under investigation, Kröll and Nüesch also examine its impact on perceived health, but find no corresponding significant effect. However, in this study, individual health is measured by a single binary outcome indicating whether the observed individuals perceive their health to be at least satisfactory. Moreover, it remains open as to whether their proposed strategy to control for individual heterogeneity adequately remedies all endogeneity concerns, given that Kröll and Nüesch merely use observations from two different years of the survey with a time difference of at least 4 years, leading to concerns of attrition bias, among others.

To summarize, the inherent relationship between WfH and health outcomes is still characterized by ambiguity despite WfH’s increasing popularity in recent years and its obvious potential effects on individual health and well-being, as outlined in Sect. “Introduction”. Many previous studies, particularly those based on observational data, struggle to apply an appropriate research design to identify causal effects. Those with appropriate identification most often focus on measures of absenteeism as indicators for objective health and ignore possible differences in perceived subjective health. Moreover, they ignore mental and social aspects of health, i.e., well-being, omit any analysis of effect mechanisms and are often based on non-representative data.

## Data and descriptives

To pursue our research question, we draw on linked employer–employee panel data from the IAB. The Linked Personnel Panel (LPP) comprises panel survey data from private-sector establishments with at least 50 employees and their employees. The first wave was conducted in 2012/2013, followed by subsequent biennial waves up to wave 4 in 2018/2019. The LPP is focused on topics of personnel economics, with questions on staff planning and recruiting, personnel development, corporate culture, variable pay, digitization, and commitment (among others). Due to its design, the LPP is representative of mid-sized and large private-sector establishments and their employees in Germany. Since pursuing our research question requires specific characteristics, in our case, tenure, experience, and the place of both work and residence to generate a commuter identifier, which are not included in the LPP survey data, we make use of its enriched version that also contains administrative information from the employment records of the Federal Employment Agency (LPP-ADIAB). The costs associated with this decision, i.e., losing observations due to the missing linkage consent of some interviewees, amount to 17.9% of the original LPP sample.

Although all four waves of the LPP cover the topic of WfH, we only use waves 2 to 4 representing the period from 2015 through 2019. This is because information from other variables we use to identify exogenous variation in the individual WfH indicator (see the subsequent section for more details) is not available in the first wave. The question underlying our WfH measure is as follows: *Do you work from home for your employer, even if only occasionally?* Hence, we focus on the effects of WfH from time to time, including part-time WfH per working day, instead of specifying an arbitrary minimum number of hours worked from home to be considered somebody who is WfH.^5^ In our sample, the prevalence of WfH increases over time from 19.8% to 28.9%. Moreover, for those WfH, the average number of hours spent on working from the own residence increases from 5.8 to 7.9 h per week. Thus, in summary, it seems that the COVID-19 pandemic and its great associated surge in the usage of WfH fueled ongoing development rather than launching it.

For our analysis, we restrict the sample to those between the ages of 20 and 65 and exclude all marginally employed workers. Regarding our outcomes of interest, we examine the current health status by means of three measures. First, we analyze an individual’s assessment of his or her current overall health, measured by a five-point Likert scale ranging from *very good* to *bad* health. We convert these five categories into five binary outcome variables which we regress separately to allow for maximum non-linearity. Second, we use the number of days being sick as reported by the surveyed employees, without limitation to days of sickness for which there is a medical certificate.^6^ Albeit also subjective in nature, this measure can be regarded as more objective than the assessment of the overall health status. Nevertheless, the number of days being sick should be interpreted with caution, as there may be differences between those WfH and those who work exclusively from their company office regarding what is reported as a sick day. We include this measure primarily for comparison purposes, as it is the most commonly used in similar studies. Third and last, we employ a well-accepted and widely used measure of well-being that is generated by means of the degree of approval to five corresponding statements, as suggested in the validated WHO-5 Well-Being Index [45]. This index employs values from 0 to 25 in its generic form, i.e., before standardization, and the higher the value, the better a person’s psychological well-being. This index was designed to depict the current mental health of an interviewee. For example, values below 13 are regarded as hinting at a potential (arising) depression.

**Table 1:** Sample means and sample mean differences by WfH status

	(1)	(2)	(3)	(4)
	All	WfH	No WfH	Difference
Panel A: outcome variables				
Very good health (dummy:1=yes)	0.170	0.206	0.160	0.046***
Good health (dummy:1=yes)	0.435	0.459	0.429	0.030**
Satisfactory health (dummy:1=yes)	0.296	0.268	0.303	– 0.036***
Poor health (dummy:1=yes)	0.077	0.054	0.084	– 0.030***
Bad health (dummy:1=yes)	0.021	0.013	0.023	– 0.011***
No. of sick days	20.846	15.799	22.304	– 6.505***
WHO-5 Well-Being Index	15.678	15.899	15.619	0.280**
Panel B: key explanatory variables				
WfH (dummy:1=yes)	0.209	1.000	0.000	1.000
Share mob. devices (in %-points)	36.160	56.382	30.829	25.552***
Panel C: covariates				
Personal characteristics:				
Female (dummy:1=yes)	0.271	0.224	0.284	– 0.060***
Migration background (dummy:1=yes)	0.070	0.051	0.075	– 0.024***
Foreigner (dummy:1=yes)	0.017	0.019	0.017	0.002
Partner (dummy:1=yes)	0.845	0.902	0.830	0.072***
Age (in years)	48.022	48.172	47.983	0.189
No. of children $< 14 yrs$	0.365	0.498	0.330	0.168***
Caregiver (dummy:1=yes)	0.123	0.108	0.127	– 0.019**
Tertiary education degree (dummy:1=yes)	0.343	0.666	0.258	0.407***
University degree (dummy:1=yes)	0.221	0.499	0.147	0.352***
Big five: thorough (5pt Likert scale)	1.489	1.530	1.478	0.051***
Big five: communicative (5pt Likert scale)	1.884	1.834	1.896	– 0.062***
Big five: rude (5pt Likert scale)	3.753	3.741	3.757	– 0.016
Big five: original (5pt Likert scale)	2.326	2.213	2.356	– 0.143***
Big five: worries (5pt Likert scale)	2.770	2.891	2.739	0.152***
Big five: forgiving (5pt Likert scale)	1.823	1.846	1.817	0.030
Big five: lazy (5pt Likert scale)	4.343	4.257	4.366	– 0.109***
Big five: outgoing (5pt Likert scale)	2.149	2.232	2.127	0.105***
Big five: artistic (5pt Likert scale)	2.778	2.749	2.785	– 0.037
Big five: nervous (5pt Likert scale)	3.513	3.640	3.480	0.160***
Big five: effective (5pt Likert scale)	1.772	1.850	1.751	0.099***
Big five: reserved (5pt Likert scale)	3.018	3.210	2.968	0.242***
Big five: considerate (5pt Likert scale)	1.784	1.853	1.765	0.088***
Big five: imaginative (5pt Likert scale)	2.478	2.464	2.482	– 0.018
Big five: relaxed (5pt Likert scale)	2.379	2.363	2.383	– 0.019
Big five: eager for knowledge (5pt Likert scale)	1.846	1.728	1.877	– 0.149***
Risk taking (10pt Likert scale)	5.637	5.864	5.577	0.287***
Employment characteristics:				
Fix-term contract (dummy:1=yes)	0.024	0.025	0.024	0.001
Part-time contract (dummy:1=yes)	0.126	0.092	0.135	– 0.043***
Shift-Work (dummy:1=yes)	0.288	0.039	0.353	– 0.313***
Actual tenure (in years)	14.276	14.224	14.290	– 0.066
Actual experience (in years)	24.081	24.070	24.084	– 0.014
No. of subordinates	9.807	30.914	4.242	26.672***
Establishment characteristics:				
No. of employees	1203.838	1846.465	1034.418	812.047***
Change of managment in last 2 yrs. (dummy:1=yes)	0.288	0.322	0.279	0.043***
Time dummies:				
Year 2015 (dummy:1=yes)	0.537	0.508	0.544	– 0.036***
Year 2017 (dummy:1=yes)	0.383	0.379	0.383	– 0.004
Year 2019 (dummy:1=yes)	0.081	0.112	0.072	0.040***
Observations	7899	1648	6251	7899

The table displays the means for the total sample (column 1), the sample of individuals who WfH (column 2), the sample of individuals who do not WfH (column 3) and the difference in means between those two subgroups (column 4). Stars for significance belong to a corresponding two sample mean t-test. The number of observations refers to each variable with the exception of the variables *No. of sick days* (3530 obs.) and *WHO-5 Well-Being Index* (7886 obs.). The notably smaller number of observations for *No. of sick days* is due to the fact that corresponding necessary information is just sampled in the LPP waves 2017 and 2019

* $$p<0.10$$, ** $$p<0.05$$, *** $$p<0.01$$

Table 1 provides an overview of the featured variables and their means, separately for all sampled employees (column 1), those WfH (column 2) and those exclusively working in their assigned office in the establishment (column 3).^7^ Within the table, the variables used are grouped into three categories. Panel A comprises our outcome variables on individual health and well-being; Panel B displays the sample means and sample mean differences for our two key explanatory variables; and Panel C includes all the covariates, from personal, employment, and establishment characteristics to the year dummies. With regard to the group differences displayed in column 4, we address only a few selected focal characteristics: first and foremost, those in our sample who are WfH report on average (and purely unconditional) better health, both regarding their subjective assessment and the comparably rather objective measure of the number of days being sick. The considerable difference in relation to the latter variable seems worth mentioning in more detail; those who work only at their company’s facilities have an average of 22.3 sick days per year, while those who work at least partially from home have only 15.8 sick days. By contrast, although they are highly significant, the sample mean differences in the five different indicators of individual subjective health are at a maximum of five percentage points. In addition, the sample mean difference of the WHO-5 Well-Being Index is rather small given the variable’s spread in terms of the standard deviation of 5.075, see Table 4.

Additionally, it is important to note that those WfH are on average employed in establishments that have provided much larger fractions of their workforce (56.4% on average) with mobile devices than their counterparts (30.8%). We take this as the first preliminary indication that this variable might in fact be a good predictor of WfH. Additionally, we observe numerous differences in the means between both groups with regard to our covariates, the most sizeable being the much larger number of subordinates and the larger establishment size for those WfH. Those individuals also have, on average, a higher level of education, more children and a partner as well as a full-time contract and are more likely to be male, without a migration background and are less likely to work in shifts. Substantial differences such as that suggesting profound positive selection into WfH emphasize the necessity for an appropriate identification strategy to uncover the causal effects of WfH.

## Empirical approach

As indicated in the last section, individuals differ systematically given their WfH status in terms of their subjective and objective health but also with respect to their socio-demographic and employment-related characteristics. We, therefore, control for those aspects in our linear model determining the effects of WfH on the different outcomes explained in the last section, which are denoted by $Y_{it}$ in the following and where we standardize the WHO-5 Well-Being Index. More precisely, ideally we would want to directly estimate:

1 $$\begin{aligned}&Y_{it} = \gamma \, \text {WfH}_{it} + \textbf{x}^{\intercal }_{1it} \, \varvec{\beta }_1 + \textbf{x}^{\intercal }_{2it} \,\nonumber \\&\quad \varvec{\beta }_2 + \textbf{x}^{\intercal }_{3et} \, \varvec{\beta }_3 + \theta _e + \theta _o + \theta _s + \theta _t + \epsilon _{it} \end{aligned}$$

where $\text {WfH}_{it}$ is a dummy variable taking the value 1 if individual *i* works from home in year *t* and 0 otherwise. Hence, the coefficient of interest is $\gamma$. $\textbf{x}_{1it}^{\intercal }$ is a vector consisting of a set of individual socio-demographic characteristics as shown in Table 1. More precisely, $\textbf{x}_{1it}^{\intercal }$ includes dummy variables for the individual’s gender, migration background, German nationality, cohabitation status, parenthood status, caregiving status, and education as reflected by indicators of a tertiary education or a university degree. It also contains a second-order polynomial for an individual’s age as well as a vector of variables describing a person’s personality (Big Five and risk aversion). $\textbf{x}_{2it}^{\intercal }$ is a vector denoting the individual’s employment-related characteristics. It comprises dummy variables for having a fixed-term work contract and working part-time or in shifts. Moreover, the individual employment history is depicted by second-order polynomial measures of tenure and experience, in which tenure is measured as years working in the same establishment and experience refers to the number of years working in a job subject to social security contributions.^8^ This vector also comprises an indicator for the number of subordinates, reflecting the observed individual’s position within the establishment’s hierarchy. $\textbf{x}_{3et}^{\intercal }$, where *e* represents the establishment level, captures different mutually exclusive dummies to control for establishment size effects, which are proxied by the number of employees per establishment.^9^$\theta _e$, $\theta _o$, $\theta _s$, and $\theta _t$ denote the establishment, occupational, occupational status, and time fixed effects (FEs). Given that hardly any establishment in our sample changes its location, $\theta _e$ additionally captures any possible regional FEs. $\theta _o$ is constructed using the first three digits of the five-digit German classification of occupations 2010 (KldB), and $\theta _s$ extracts the information from the fifth digit, i.e., the level of requirement.^10^ Three different time dummies representing the different waves of the LPP are represented by $\theta _t$. Lastly, the error term $\epsilon _{it}$ captures the additional heterogeneity that cannot be explained by our set of regressors.

However, even after conditioning on the rich set of covariates depicted by Eq. 1, $\gamma$ might not represent the causal effect of WfH on the different health and well-being outcomes. As mentioned above, Table 1 indicates a positive selection. Hence, there might be some unobservable factors that are related to both the individual decision of WfH as well as the health and well-being outcomes. Given our set of different FEs in Eq. 1, we can rule out any spurious effects of time-invariant characteristics at the establishment, occupation, and occupational status levels. Hence, $\gamma$ is actually identified by those individuals with differences in the WfH status while being employed in the same establishment, occupation and occupational status, i.e., exploiting intertemporal variation in WfH at the individual level and intralevel variation in WfH at the above-mentioned levels. Unfortunately, we are not able to consider individual FEs in Eq. 1 to exclude any confounding time-invariant factors at the individual level, such as time-invariant preferences. This drawback is due to the limited time dimension of our sample, which merely exploits information from three different LPP waves (see Section “Data and descriptives”) and the considerably low within-variation in the WfH status at the individual level (approx. 9%) preventing identification at this level.^11^ However, to at least mitigate this shortcoming, aside from general individual characteristics, we also control for personality traits, that have been shown to potentially help reducing selection biases on the individual level [47].

Despite controlling for the different time-varying observables as well as FEs, we cannot credibly rule out any additional endogeneity caused by time-varying unobservable variables at the individual, establishment, occupational or occupational status level, which are contained in $\epsilon _{it}$. For instance, family or more general private responsibilities which are not (adequately) captured by our controls of the number of children younger than 14 years old or caregiving could have an impact on the decision of WfH but also affecting the own health. The same applies to the location of living and the accommodation. In addition, identification could also be threatened by reverse causality issues, arguing that relatively more sick individuals, such as those with chronic diseases, might be tempted to make use of WfH more frequently.

To address these concerns, we apply an IV strategy to be able to claim our estimated effects of WfH to be causal. Our instrument is required to explain the individual’s decision of WfH while having no direct impact on one of the subjective and objective health and well-being outcomes. To fulfill this necessary requirement, we exploit information that is sampled at the establishment level. More precisely, we make use of the information on the share of employees with and without managerial responsibility per establishment that is equipped with mobile devices capable of establishing an internet connection via the mobile network. Mobile devices can be smart phones, tablet computers or notebooks. We combine this information with the responsibility of the observed individuals within their establishments. Our instrument equals the equipped share of employees with managerial responsibility for those observed individuals being in a leadership position and is equal to the equipped share of employees without managerial responsibility for all other employees.^12^ Given the linearity, we estimate Eq. 1 by means of two-stage least squares (2SLS). More formally, Eq. 2 represents our first-stage estimation, where $\text {smd}_{mt}$ denotes the instrument varying at a within-establishment level *m* for a given point of time while $\psi _{\cdot }$ represents the above-mentioned different levels of FEs and $\nu _{it}$ denotes the error term of this first stage.

2 $$\begin{aligned} \text {WfH}_{it}&= \pi \, \text {smd}_{mt} + \textbf{x}^{\intercal }_{1it} \, \varvec{\alpha }_1 + \textbf{x}^{\intercal }_{2it} \, \varvec{\alpha }_2\nonumber \\&\quad + \textbf{x}^{\intercal }_{3et} \, \varvec{\alpha }_3 + \psi _e + \psi _o + \psi _s + \psi _t + \nu _{it} \end{aligned}$$

**Fig. 2:**
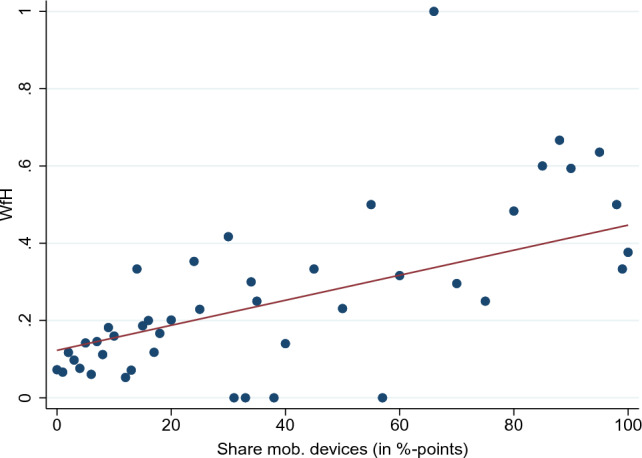
WfH status depends on the share of employees equipped with mobile devices. Note: The red line plots a linear fit to the data of grouped individual observations of WfH and the instrument of the share of mobile devices

Figure 2 plots the relationship between our instrument and the endogenous variable WfH. It shows the proportion of grouped individuals WfH by percentiles of the share of employees equipped with mobile devices, as outlined in the last paragraph. The fitted line shows a distinct positive relationship despite the outliers for some specific levels of our instrument where every group member is either exclusively WfH or exclusively working from the office. In particular, our chosen instrument seems to be a good predictor for those groups of individuals rarely WfH. For individuals working in establishments in which a large share of employees is equipped with mobile devices, Fig. 2 shows that the decision of WfH is subject to individual choice. In other words, even in observed individuals’ establishments where almost everyone is equipped with a mobile device, not every employee is deciding WfH. However, the unconditional correlation between both variables of $\rho \approx 0.3$ is still quite large.

Despite our approach of explaining the exogenous within-variation of WfH by means of an instrument as described above, some identification threats could still occur. Most prominently, our chosen instrument facilitates not only WfH but also working at places other than the office or the own residence. This behavior is known as mobile working and subsumes working during commuting times, in particular when traveling by train for long distances, on business trips or even when on holiday. It could confound our WfH estimates if mobile working has a direct impact on health and well-being outcomes, which seems to be plausible. Unfortunately, we are not able to control for mobile working directly since this behavior is not addressed in the LPP survey. However, given data from the American Time Use Survey (ATUS) of the Bureau of Labor Statistics (BLS), it seems unlikely that mobile working affects our identification strategy.^13^ According to own calculations reported in Table 5, US citizens have spent more than 92.6% of their working time at home or in the office for all 3 years under investigation. As shown in Panel B of Table 5, this is also true for a sample mimicking our LPP sample restrictions. Unfortunately, we are not able to present some corresponding evidence for Germany since the latest German Time Use Survey (GTUS) is from 2012 to 2013, but we have no reason to believe that this pattern should be systematically different between the US and the German labor market.

Related to the potential threat of mobile working, different amounts of screen time among those individuals WfH and those who do not could confound our estimates, given that screen time might have an impact on individual health. This would be true if those individuals WfH would generally exhibit different amounts of screen time in comparison to those who are not WfH and those differences could not be explained at either the establishment, occupation or occupational status level. While we are not able to control for this aspect in our regression since the information of screen time is merely sampled in the fourth wave of the LPP, we are able to provide some descriptive evidence. Table 6 shows the share of daily hours spent on different screen-time-related activities of the total number of daily working hours for the two groups. More precisely, it shows that for four out of six activities, the share of a group of individuals WfH is not larger than that of a control group consisting of individuals who could work from home job-wise and who are allowed by the establishment to do so but decide not to.^14^

The remaining threats to identification are related to intertemporal variation at one of the different FE levels. Most prominent, unobservable demand shocks leading to an increase in workload could affect our estimates. Therefore, we additionally control for individual overtime in a robustness check and find similar results in comparison to those presented in the next section.^15^

## Results

Table 2 displays the effects of WfH on the measures of individual subjective health. It is worth noting that according to the proposed test by Olea and Pflueger [50] on excluded instruments for a linear regression with clustered standard errors, our chosen instrument turns out to be sufficiently strong to explain the exogenous variation in WfH. As shown in Panel B of Table 2, this test yields an Effective F-statistic of approx. 52.5, which is far larger than the critical value proposed by Olea and Pflueger of 23.1, corresponding to a 10% worst-case bias in the 2SLS estimates. The positive first-stage coefficient, i.e., $\hat{\pi }$ of Eq. 2, is also consistent with our expectation showing that an increase in the share of employees equipped with mobile devices leads to an increase in the individual likelihood of WfH.

The 2SLS estimation displayed in Table 2 yields a positive and highly significant effect for the impact of WfH on the most favorable indicator of the five assessment categories (very good), which is more or less balanced by an (almost) equally large but negative effect on the indicator of satisfactory health. In more detail, the effects suggest that WfH reduces the likelihood of having satisfactory health by 31 percentage points and increases that of very good health by 29 percentage points. Taken together, both significant effects suggest that WfH improves subjective overall health. Considering the insignificant yet pattern-confirming effects of the other health categories, positive for good health and negative for poor and bad health, respectively, further supports this interpretation. This result becomes even clearer when also taking into account the skewed distribution of subjective health. Comparably few employees, i.e., fewer than 10%, assigned themselves poor or bad health, making it difficult to estimate significant effects with our sample size.

**Table 2:** Effect of WfH on individual subjective health

	Panel A					Panel B
Outcome:	Very good	Good	Satisfactory	Poor	Bad	WfH
	2SLS 2nd St.	2SLS 2nd St.	2SLS 2nd St.	2SLS 2nd St.	2SLS 2nd St.	2SLS 1st St.
WfH (dummy:1=yes)	0.289**	0.0993	– 0.313*	– 0.00958	– 0.0658	
	(0.132)	(0.173)	(0.161)	(0.0900)	(0.0489)	
Share mob. devices						0.00105***
						(0.000145)
Establishment FE	Yes	Yes	Yes	Yes	Yes	Yes
Occupation FE	Yes	Yes	Yes	Yes	Yes	Yes
Occupational status FE	Yes	Yes	Yes	Yes	Yes	Yes
Year FE	Yes	Yes	Yes	Yes	Yes	Yes
Observations	7899	7899	7899	7899	7899	7899
Effective F-statistic						52.56

Panel A shows the estimated coefficients of the 2SLS second-stage regressions of Eq. 1. Panel B displays the estimated coefficients of the 2SLS first-stage regression of Eq. 2. Control variables are included as indicated in the respective equations. Standard errors reported in parentheses are clustered at the individual level for all regressions. The Effective F-statistic refers to Olea and Pflueger [50]. See Table 7 for the complete regression table

* $$p<0.10$$, ** $$p<0.05$$, *** $$p<0.01$$

Turning to the more objective and most frequently analyzed measure of individual health in this context, the number of days being sick, our 2SLS estimation reveals a negative but insignificant effect (see Table 3). The sign of the effect appears to be fairly stable, since over the different specifications we test, it always turns out negative.^16^ In this respect, disregarding its insignificance, the estimated effect is consistent with other research on this topic as discussed in the literature review; see Sect. “Literature”. Its large value of approximately minus fifteen may seem enormous at first glance; however, the substantial difference between the means of both groups displayed in Table 1 of almost 6.5 days puts it into perspective. Also, given the large standard error, the effect is simply imprecisely estimated, despite the sufficiently strong first stage (Effective F-statistic of approx. 26.4). This may be caused by greater heterogeneity among employees WfH regarding their behavior of reporting sick. Given a certain level of illness that prevents (most of) those not WfH to call in sick and avoid the commute, among those WfH some may still decide to work, although with lower intensity/efficiency (answer mails, attend calls, etc.), as has been shown by Bloom et al. [12].

Table 3 also reports the results of our analysis on the effects of WfH on well-being as another more specific health measure. Our preferred specification based on the 2SLS estimation and the standardized version of the outcome variable yields a positive significant effect, implying that WfH increases well-being. Given the substantial effect of 0.64 implying an increase in well-being by 0.64 standard deviations, the effect appears to be not only statistically but also economically significant.

**Table 3:** Effect of WfH on individual objective health and well-being

Outcome:	No. of sick days		WHO-5 Well-Being Index *standardized*	
	2SLS		2SLS	
	2nd St.	1st St.	2nd St.	1st St.
WfH (dummy:1=yes)	– 14.61		0.636*	
	(13.62)		(0.341)	
Share mob. devices		0.00118***		0.00106***
		(0.000230)		(0.000146)
Establishment FE	Yes	Yes	Yes	Yes
Occupation FE	Yes	Yes	Yes	Yes
Occupational status FE	Yes	Yes	Yes	Yes
Year FE	Yes	Yes	Yes	Yes
Observations	3530	3530	7850	7850
Effective F-statistic	26.371	26.371	52.905	52.905

This table shows the estimated coefficients of the 2SLS second-stage regressions of Eq. 1 and the estimated coefficients of the 2SLS first-stage regression of Eq. 2 for the outcome of number of sick days as well as the standardized WHO-5 Well-Being Index. Control variables are included as indicated in the respective equations. For the number of sick days specification, a measure of subjective health is additionally included. Standard errors reported in parentheses are clustered at the individual level for all regressions. The Effective F-statistic refers to Olea and Pflueger 50]. See Table 8 for the complete regression table

* $$p<0.10$$, ** $$p<0.05$$, *** $$p<0.01$$

Taken together, our results on the impact of WfH on individual subjective and objective health and well-being suggest a beneficial effect of pursuing work while staying at home.^17^ This effect appears to be most pronounced for psychological well-being. However, considering the numerous potential channels discussed in the motivation of this paper, there may be differential effects through certain characteristics. In this regard, Table 9 in the appendix provides an overview of the effect heterogeneities with respect to gender, parenthood, age, and commuting status.^18^ The differences between the coefficients imply that the beneficial effects of WfH on health and well-being tend to be more pronounced for men. Regarding age, our separate estimations for three age groups reveal that the effects are strongest for the middle-aged group from 35 to 50 years old, indicating the role of WfH in a stage of life that is often the most heavily influenced by both career and family (formation). Finally, because the effects for those not commuting cannot be interpreted given the missing instrument’s relevance in this specification, we cannot contrast them with those of the commuting group. We can only observe that the beneficial effects of WfH on overall health and well-being estimated based on the full sample are not present when solely looking at commuters.^19^

## Conclusion

There are several perspectives and facets regarding the phenomenon of WfH, in which interest has surged in the wake of the COVID-19 pandemic. While most contributions to the literature to date focus on standard work-related aspects such as working hours or wages, this paper addresses the under-researched effects on health and well-being. Using unique linked employer–employee panel data from Germany and employing an IV approach to estimate causal effects, we find evidence of a beneficial impact of WfH on both individual health and well-being. While there seems to be no effect on the number of days being sick despite its negative (but insignificant) coefficient, WfH increases subjective overall health and, in particular, well-being. Given the pronounced impact on the WHO-5 Well-Being Index covering psychological health by a magnitude of 0.64 standard deviations, the comparably small effect on subjective health may suggest that there is no or only a modest impact on physical health. Hence, the overall health assessment seems to be (primarily) driven by its mental (well-being) component.

Taken together, our results imply that it may be beneficial for companies to allow WfH for those with suitable occupations. While employers may not be awarded with a significant reduction in costly sick days, they may obtain (mentally) healthier workers, which according to the literature (e.g., Bubonya et al. [51]) might in turn have other beneficial implications on commitment, engagement, and productivity. It is also worth noting that WfH is usually not something that is (forcefully) applied to employees. Except during the pandemic, it is mostly the workers’ wishes that ultimately determine whether a firm-provided possibility of WfH is actually put to use. Consequently, the benefits from providing the prerequisites for WfH may be even larger since it is mostly used by employees who expect to profit from WfH.

Nevertheless, there are still many aspects related to the impact of WfH on health and well-being that we could not address within the scope of this paper and using the underlying data. First and foremost, it is important to shed more light on the several channels of this relationship, for which more detailed analyses of potential heterogeneities are required. Identifying the channels that are mostly responsible will be key to understanding which groups benefit the most and which might need supporting measures to exploit a beneficial impact of WfH on health. Data from time-use surveys could help to identify corresponding effects. Another possibly promising approach would be to make use of detailed data on the period of the current COVID-19 pandemic, in which not only a positive selection of individuals self-selected into WfH but also many others did despite their opposition toward WfH, which was put on hold during the pandemic, be it for self-protection or for the greater good. This setting could help not only to verify our findings but also to examine whether nowadays much higher average intensity of WfH leads to even stronger positive effects on health and well-being or whether the relationship is in fact non-linear, with potential negative effects outweighing positive effects with increasing intensity of use (i.e., an inversely U-shaped relationship).

## Appendix

See Tables 4, 5, 6, 7, 8, 9

**Table 4:** Summary statistics on LPP sample

	(1)	(2)	(3)	(4)
	Mean	SD	Min	Max
Panel A: outcome variables				
Very good health (dummy:1=yes)	0.170	0.376	0.000	1.000
Good health (dummy:1=yes)	0.435	0.496	0.000	1.000
Satisfactory health (dummy:1=yes)	0.296	0.457	0.000	1.000
Poor health (dummy:1=yes)	0.077	0.267	0.000	1.000
Bad health (dummy:1=yes)	0.021	0.144	0.000	1.000
No. of sick days	20.846	31.973	0.000	279.000
WHO-5 Well-Being Index	15.678	5.075	0.000	25.000
Panel B: key explanatory variables				
WfH (dummy:1=yes)	0.209	0.406	0.000	1.000
Share mob. devices (in %-points)	36.160	39.488	0.000	100.000
Panel C: covariates				
Personal characteristics:				
Female (dummy:1=yes)	0.271	0.445	0.000	1.000
Migration background (dummy:1=yes)	0.070	0.255	0.000	1.000
Foreigner (dummy:1=yes)	0.017	0.130	0.000	1.000
Partner (dummy:1=yes)	0.845	0.362	0.000	1.000
Age (in years)	48.022	9.879	20.000	65.000
No. of children $< 14$ yrs	0.365	0.730	0.000	5.000
Caregiver (dummy:1=yes)	0.123	0.328	0.000	1.000
Tertiary education degree (dummy:1=yes)	0.343	0.475	0.000	1.000
University degree (dummy:1=yes)	0.221	0.415	0.000	1.000
Big five: thorough (5pt Likert scale)	1.489	0.570	1.000	5.000
Big five: communicative (5pt Likert scale)	1.884	0.868	1.000	5.000
Big five: rude (5pt Likert scale)	3.753	1.084	1.000	5.000
Big five: original (5pt Likert scale)	2.326	0.859	1.000	5.000
Big five: worries (5pt Likert scale)	2.770	1.136	1.000	5.000
Big five: forgiving (5pt Likert scale)	1.823	0.722	1.000	5.000
Big five: lazy (5pt Likert scale)	4.343	0.792	1.000	5.000
Big five: outgoing (5pt Likert scale)	2.149	0.914	1.000	5.000
Big five: artistic (5pt Likert scale)	2.778	1.164	1.000	5.000
Big five: nervous (5pt Likert scale)	3.513	1.074	1.000	5.000
Big five: effective (5pt Likert scale)	1.772	0.569	1.000	5.000
Big five: reserved (5pt Likert scale)	3.018	1.119	1.000	5.000
Big five: considerate (5pt Likert scale)	1.784	0.625	1.000	5.000
Big five: imaginative (5pt Likert scale)	2.478	1.027	1.000	5.000
Big five: relaxed (5pt Likert scale)	2.379	0.924	1.000	5.000
Big five: eager for knowledge (5pt Likert scale)	1.846	0.735	1.000	5.000
Risk taking (10pt Likert scale)	5.637	1.800	0.000	10.000
Employment characteristics:				
Fix-term contract (dummy:1=yes)	0.024	0.154	0.000	1.000
Part-time contract (dummy:1=yes)	0.126	0.332	0.000	1.000
Shift-Work (dummy:1=yes)	0.288	0.453	0.000	1.000
Actual tenure (in years)	14.276	9.463	0.140	43.030
Actual experience (in years)	24.081	9.449	1.279	63.411
No. of subordinates	9.807	180.637	0.000	15000.000
Establishment characteristics:				
No. of employees	1203.838	4456.721	2.000	44539.000
Change of managment in last 2 yrs. (dummy:1=yes)	0.288	0.453	0.000	1.000
Time dummies:				
Year 2015 (dummy:1=yes)	0.537	0.499	0.000	1.000
Year 2017 (dummy:1=yes)	0.383	0.486	0.000	1.000
Year 2019 (dummy:1=yes)	0.081	0.272	0.000	1.000
Observations	7899	7899	7899	7899

The number of observations refers to each variable with the exception of the variables *No. of sick days* (3530 obs.) and *WHO-5 Well-Being Index* (7886 obs.). The notably smaller number of observations for *No. of sick days* is due to the fact that corresponding necessary information is just sampled in the LPP waves 2017 and 2019

**Table 5:** Summary statistics on minutes spent on working activities per day - ATUS sample

	(1)	(2)	(3)
Year	2015	2017	2019
	Mean / median	Mean / median	Mean / median
Panel A: full sample			
Working time at office	359.7	365.7	352.0
	450.0	450.0	445.0
Working time at home	53.2	48.5	54.1
	0.0	0.0	0.0
Total working time	429.7	437.2	428.8
	470.0	480.0	472.0
Share WT (home or office / total) in %	94.5	92.8	93.4
	100.0	100.0	100.0
Observations	3904	3588	3265
Panel B: restricted sample			
Working time at office	396.2	398.7	380.0
	470.0	471.0	460.0
Working time at home	40.0	38.4	45.0
	0.0	0.0	0.0
Total working time	449.1	456.6	444.1
	480.0	484.0	480.0
Share WT (home or office / total) in %	95.8	93.8	94.5
	100.0	100.0	100.0
Observations	2568	2375	2173

The restricted sample excludes all individuals younger than 20 or older than 65 years old. Moreover, it excludes all individuals not working in the private sector. Therefore, it is more similar to the main sample of investigation presented in Sect. “Data and descriptives”. Both daily minutes working in the main job and working in some other job are considered in this calculation. Other possible locations to work sampled in the ATUS besides the own home or office are: someone else’s home; restaurant or bar; place of worship; grocery store; other store/mall; school; outdoors away from home; library; other place; car, truck, or motorcycle (driver); car, truck, or motorcycle (passenger); walking; bus; subway/train; bicycle; boat/ferry; taxi/limousine service; airplane; other mode of transportation; bank; gym/health club; post office; unspecified place and unspecified mode of transportation

**Table 6:** Sample shares of actual daily working hours spent on screen time related working activities of total daily working hours and sample shares differences by WfH status - LPP sample Wave 4

	(1)	(2)	(3)	(4)
	All	WfH	No WfH	Difference
% Meetings by telephone or internet calls	10.6	13.0	6.8	6.2***
% Writing and reading message	18.2	20.4	14.7	5.7***
% Writing or revising texts on computer, laptop or tablet	16.9	17.6	15.8	1.8
% Entering or processing data on a computer, laptop or tablet	17.2	15.0	20.6	$-$5.6**
% Researching and collecting information online	5.0	5.1	4.8	0.4
% Programming	3.4	3.2	3.7	– 0.5
Observations	401	242	159	401

The table displays the shares for the total sample (column 1), the sample of individuals who WfH (column 2), the sample of individuals who do not WfH but could do so job-wise and by the permission of the employer (column 3) and the difference in shares between those two subgroups (column 4). Stars for significance belong to a corresponding two sample mean *t* test. Information on the employers permission of WfH and whether the job tasks of the individuals allow WfH are taken from LPP Wave 3

* $$p<0.10$$, ** $$p<0.05$$, *** $$p<0.01$$

**Table 7:** Effect of WfH on individual subjective health

	Panel A					Panel B
Outcome:	Very good	Good	Satisfactory	Poor	Bad	WfH
	2SLS 2nd St.	2SLS 2nd St.	2SLS 2nd St.	2SLS 2nd St.	2SLS 2nd St.	2SLS 1st St.
WfH (dummy:1=yes)	0.289**	0.0993	– 0.313*	– 0.00958	– 0.0658	
	(0.132)	(0.173)	(0.161)	(0.0900)	(0.0489)	
Share mob. devices						0.00105***
						(0.000145)
Female (dummy:1=yes)	0.0288*	0.0126	– 0.0273	– 0.00288	– 0.0113*	– 0.0566***
	(0.0170)	(0.0221)	(0.0208)	(0.0118)	(0.00635)	(0.0153)
Migration background (dummy:1=yes)	0.0117	0.0116	– 0.0305	0.00841	– 0.00133	– 0.00852
	(0.0218)	(0.0266)	(0.0245)	(0.0156)	(0.00759)	(0.0178)
Foreigner (dummy:1=yes)	– 0.0309	0.0449	0.0200	– 0.0388	0.00483	0.00962
	(0.0384)	(0.0485)	(0.0441)	(0.0251)	(0.0153)	(0.0368)
Partner (dummy:1=yes)	– 0.00809	0.0368*	– 0.0234	– 0.00508	– 0.000209	0.0390***
	(0.0147)	(0.0191)	(0.0181)	(0.0104)	(0.00612)	(0.0123)
Age (in years)	– 0.0207***	0.00513	0.0111*	– 0.000953	0.00548***	0.00851*
	(0.00560)	(0.00695)	(0.00625)	(0.00370)	(0.00190)	(0.00494)
Age^2^ (in years^2^)	0.000167***	– 0.0000976	– 0.0000694	0.0000442	– 0.0000441**	– 0.000109**
	(0.0000588)	(0.0000741)	(0.0000673)	(0.0000399)	(0.0000203)	(0.0000519)
Parent (dummy:1=yes)	0.0143	0.00285	– 0.0116	– 0.00572	0.000165	0.0311**
	(0.0129)	(0.0167)	(0.0154)	(0.00839)	(0.00517)	(0.0126)
Caregiver (dummy:1=yes)	0.00127	– 0.0271	– 0.0170	0.0326***	0.0103	– 0.00405
	(0.0127)	(0.0174)	(0.0170)	(0.0109)	(0.00676)	(0.0128)
Tertiary education degree (dummy:1=yes)	– 0.0210	0.00247	0.00234	0.00899	0.00721	0.0573***
	(0.0162)	(0.0210)	(0.0195)	(0.0108)	(0.00599)	(0.0158)
University Degree (dummy:1=yes)	0.0549**	– 0.0223	– 0.000581	– 0.0318**	– 0.000133	0.0828***
	(0.0216)	(0.0272)	(0.0259)	(0.0139)	(0.00758)	(0.0203)
Risk Taking (10pt Likert scale)	– 0.00349	0.00124	0.000344	– 0.0000843	0.00199*	0.00747***
	(0.00303)	(0.00386)	(0.00359)	(0.00208)	(0.00113)	(0.00281)
Fix-term contract (dummy:1=yes)	0.0279	– 0.0280	– 0.0194	0.0146	0.00492	0.0105
	(0.0310)	(0.0382)	(0.0363)	(0.0223)	(0.0102)	(0.0304)
Part-time contract (dummy:1=yes)	– 0.00365	0.0116	0.00168	– 0.00261	– 0.00706	– 0.0315*
	(0.0178)	(0.0235)	(0.0225)	(0.0132)	(0.00775)	(0.0169)
Shift-Work (dummy:1=yes)	– 0.00524	0.00322	0.0156	– 0.0115	– 0.00207	– 0.0616***
	(0.0160)	(0.0212)	(0.0197)	(0.0118)	(0.00641)	(0.0110)
Actual tenure (in years)	– 0.00460**	– 0.00149	0.00655**	– 0.00122	0.000762	0.00187
	(0.00212)	(0.00277)	(0.00265)	(0.00151)	(0.000795)	(0.00212)
Actual tenure^2^ (in years^2^)	0.0000743	0.0000748	– 0.000167**	0.0000400	– 0.0000223	– 0.0000712
	(0.0000542)	(0.0000738)	(0.0000706)	(0.0000416)	(0.0000221)	(0.0000560)
Actual experience (in years)	– 0.00342	0.00696	– 0.000171	– 0.00119	– 0.00218	0.00322
	(0.00343)	(0.00458)	(0.00424)	(0.00248)	(0.00134)	(0.00336)
Actual experience^2^ (in years^2^)	0.0000690	– 0.000101	0.0000110	– 0.00000538	0.0000263	– 0.0000514
	(0.0000581)	(0.0000805)	(0.0000757)	(0.0000441)	(0.0000233)	(0.0000564)
No. of subordinates (in hundreds)	– 0.000509	0.000536	0.000348	– 0.000372	– 0.00000339	0.00332
	(0.00104)	(0.00290)	(0.00246)	(0.000445)	(0.000315)	(0.00295)
100 <= Empl. < 200 (dummy:1=yes)	0.00860	0.0999	– 0.149*	0.0302	0.0104	0.0121
	(0.0679)	(0.0738)	(0.0797)	(0.0356)	(0.0356)	(0.0535)
200 <= Empl. < 500 (dummy:1=yes)	0.0491	0.0578	– 0.179*	0.0620	0.0102	0.0169
	(0.0766)	(0.0957)	(0.0989)	(0.0499)	(0.0383)	(0.0628)
500 <= Empl. < 1.000 (dummy:1=yes)	0.0763	0.0857	– 0.269**	0.111**	– 0.00389	– 0.00941
	(0.0925)	(0.120)	(0.120)	(0.0556)	(0.0430)	(0.0761)
1.000 > Empl. (dummy:1=yes)	0.182	– 0.0785	– 0.233	0.0984	0.0315	– 0.0511
	(0.122)	(0.157)	(0.162)	(0.0962)	(0.0576)	(0.117)
Change of managment in last 2 yrs. (dummy:1=yes)	– 0.00953	0.0143	0.0293	– 0.00951	0.00410	0.0153
	(0.0150)	(0.0207)	(0.0192)	(0.0107)	(0.00572)	(0.0150)
Big five individual controls	Yes	Yes	Yes	Yes	Yes	Yes
Establishment FE	Yes	Yes	Yes	Yes	Yes	Yes
Occupation FE	Yes	Yes	Yes	Yes	Yes	Yes
Occupational status FE	Yes	Yes	Yes	Yes	Yes	Yes
Year FE	Yes	Yes	Yes	Yes	Yes	Yes
Observations	7899	7899	7899	7899	7899	7899
Effective F-statistic						52.56

Panel A shows the estimated coefficients of the 2SLS second stage regressions of Eq. 1. Panel B displays the estimated coefficients of the 2SLS first stage regression of Eq. 2. Control variables are included as indicated in the respective equations. Standard errors reported in parentheses are clustered at the individual level for all regressions. The Effective F-statistic refers to Olea and Pflueger [50]. Coefficients of the Big Five Individual Controls are available on request

* $$p<0.10$$, ** $$p<0.05$$, *** $$p<0.01$$

**Table 8:** Effect of WfH on individual objective health and well-being

Outcome:	No. of sick days		WHO-5 Well-Being Index *standardized*	
	2SLS		2SLS	
	2nd St.	1st St.	2nd St.	1st St.
WfH (dummy:1=yes)	– 14.61		0.636*	
	(13.62)		(0.341)	
Share mob. devices		0.00118***		0.00106***
		(0.000230)		(0.000146)
Female (dummy:1=yes)	0.456	– 0.0369	0.0301	– 0.0545***
	(1.837)	(0.0243)	(0.0436)	(0.0155)
Migration background (dummy:1=yes)	2.863	0.0370	– 0.0450	– 0.00812
	(2.515)	(0.0294)	(0.0546)	(0.0179)
Foreigner (dummy:1=yes)	2.069	0.0275	0.0356	0.00980
	(5.170)	(0.0608)	(0.109)	(0.0368)
Partner (dummy:1=yes)	– 0.112	0.0479**	0.0624	0.0394***
	(1.608)	(0.0194)	(0.0398)	(0.0123)
Age (in years)	– 0.489	0.00960	– 0.0304**	0.00896*
	(0.572)	(0.00864)	(0.0132)	(0.00496)
Age^2^ (in years^2^)	0.00631	– 0.000120	0.000413***	– 0.000114**
	(0.00604)	(0.0000898)	(0.000140)	(0.0000521)
Parent (dummy:1=yes)	2.313	0.0543***	– 0.0514	0.0317**
	(1.418)	(0.0209)	(0.0318)	(0.0126)
Caregiver (dummy:1=yes)	1.941	– 0.00760	– 0.162***	– 0.00351
	(1.634)	(0.0219)	(0.0373)	(0.0130)
Tertiary education degree (dummy:1=yes)	1.908	0.0718***	– 0.0991**	0.0573***
	(1.734)	(0.0238)	(0.0406)	(0.0159)
University degree (dummy:1=yes)	– 2.830	0.102***	– 0.00323	0.0825***
	(2.015)	(0.0306)	(0.0508)	(0.0204)
Risk taking (Likert scale)	0.482	0.0121***	0.0186**	0.00723**
	(0.369)	(0.00442)	(0.00794)	(0.00282)
Fix-term contract (dummy:1=yes)	4.498	0.0295	– 0.0698	0.0132
	(3.220)	(0.0588)	(0.0763)	(0.0304)
Part-time contract (dummy:1=yes)	– 1.504	– 0.0533*	0.0230	– 0.0336**
	(2.163)	(0.0285)	(0.0476)	(0.0170)
Shift-work (dummy:1=yes)	1.627	– 0.0622***	– 0.0223	– 0.0621***
	(1.883)	(0.0192)	(0.0425)	(0.0110)
Actual tenure (in years)	– 0.117	0.00456	– 0.0108**	0.00186
	(0.238)	(0.00336)	(0.00549)	(0.00213)
Actual tenure^2^ (in years^2^)	0.000652	– 0.000139	0.000208	– 0.0000702
	(0.00621)	(0.0000870)	(0.000146)	(0.0000563)
Actual experience (in years)	0.0501	0.00387	– 0.00157	0.00296
	(0.375)	(0.00577)	(0.00908)	(0.00337)
Actual experience^2^ (in years^2^)	– 0.00183	– 0.0000669	0.0000588	– 0.0000464
	(0.00644)	(0.0000963)	(0.000160)	(0.0000565)
No. of subordinates (in hundreds)	– 0.147***	0.00221	0.00559*	0.00329
	(0.0537)	(0.00224)	(0.00306)	(0.00288)
100 <= Empl. < 200 (dummy:1=yes)	– 11.22*	– 0.277	– 0.0726	0.0118
	(6.779)	(0.276)	(0.174)	(0.0535)
200 <= Empl. < 500 (dummy:1=yes)	– 14.90	– 0.493*	– 0.222	0.0139
	(10.91)	(0.300)	(0.216)	(0.0630)
500 <= Empl. < 1.000 (dummy:1=yes)	– 7.975	– 0.533*	– 0.173	– 0.0133
	(13.18)	(0.307)	(0.248)	(0.0763)
1.000 > Empl. (dummy:1=yes)	48.46	– 0.518*	– 0.323	– 0.0605
	(48.55)	(0.312)	(0.326)	(0.118)
Change of managment in last 2 yrs. (dummy:1=yes)	3.316*	0.0408	– 0.0781*	0.0156
	(2.001)	(0.0298)	(0.0408)	(0.0151)
Very good health (dummy:1=yes)	– 12.50***	– 0.00463		
	(1.478)	(0.0236)		
Good health (dummy:1=yes)	– 8.133***	– 0.0394**		
	(1.304)	(0.0163)		
Poor health (dummy:1=yes)	17.95***	– 0.0141		
	(2.954)	(0.0250)		
Bad health (dummy:1=yes)	34.10***	0.00997		
	(5.738)	(0.0576)		
Big five individual controls	Yes	Yes	Yes	Yes
Establishment FE	Yes	Yes	Yes	Yes
Occupation FE	Yes	Yes	Yes	Yes
Occupational FE	Yes	Yes	Yes	Yes
Year FE	Yes	Yes	Yes	Yes
Observations	3530	3530	7850	7850
Effective F-statistic	26.371	26.371	52.905	52.905

This table shows the estimated coefficients of the 2SLS second stage regressions of Eq. 1 and the estimated coefficients of the 2SLS first stage regression of Eq. 2 for the outcome of number of sick days as well as the standardized WHO-5 Well-Being Index. Control variables are included as indicated in the respective equations. For the number of sick days specification, a measure of subjective health is additionally included. Standard errors reported in parentheses are clustered at the individual level for all regressions. The Effective F-statistic refers to Olea and Pflueger [50]. Coefficients of the big five individual controls are available on request

* $$p<0.10$$, ** $$p<0.05$$, *** $$p<0.01$$

**Table 9:** Effect of WfH - Heterogeneity analysis

	Gender		Parenthood		Age			Commuting	
	Male	Female	Childless	Parent	Age < 35	35 <= Age < 50	Age >= 50	Commuter	No Comm.
Very good health:									
WfH (dummy:1=yes)	0.373**	**0.153**	0.175	0.601*	– **0.908**	0.694**	0.155	0.214	**0.568**
	(0.159)	**(0.325)**	(0.151)	**(0.327)**	**(1.161)**	(0.276)	(0.166)	(0.157)	**(1.444)**
Observations	5755	**2144**	6027	**1872**	**1006**	2763	4130	5445	**1452**
Effective F-statistic	37.85	**5.761**	35.08	**8.987**	**0.875**	13.21	24.51	32.32	**0.477**
Good health:									
WfH (dummy:1=yes)	0.104	**0.0607**	0.125	$-$0.555	**0.784**	$-$0.204	0.0119	$-$0.0443	**1.774**
	(0.201)	**(0.498)**	(0.213)	**(0.372)**	**(1.232)**	(0.304)	(0.247)	(0.209)	**(2.377)**
Observations	5755	**2144**	6027	**1872**	**1006**	2763	4130	5445	**1452**
Effective F-statistic	37.85	**5.761**	35.08	**8.987**	**0.875**	13.21	24.51	32.32	**0.477**
Satisfactory health:									
WfH (dummy:1=yes)	$-$0.388**	− **0.294**	$-$0.199	**0.0591**	**0.419**	$-$0.406	$-$0.243	$-$0.144	– **1.616**
	(0.191)	**(0.478)**	(0.200)	**(0.295)**	**(0.877)**	(0.272)	(0.244)	(0.190)	**(2.303)**
Observations	5755	**2144**	6027	**1872**	**1006**	2763	4130	5445	**1452**
Effective F-statistic	37.85	**5.761**	35.08	**8.987**	**0.875**	13.21	24.51	32.32	**0.477**
Poor health:									
WfH (dummy:1=yes)	$-$0.0247	**0.115**	$-$0.0464	**0.0708**	– **0.142**	$-$0.0243	0.167	0.0421	**0.0271**
	(0.101)	**(0.309)**	(0.119)	**(0.157)**	**(0.458)**	(0.142)	(0.151)	(0.112)	**(0.819)**
Observations	5755	**2144**	6027	**1872**	**1006**	2763	4130	5445	**1452**
Effective F-statistic	37.85	**5.761**	35.08	**8.987**	**0.875**	13.21	24.51	32.32	**0.477**
Bad health:									
WfH (dummy:1=yes)	– 0.0637	– **0.0353**	– 0.0548	– 0.176*	– **0.154**	– 0.0598	– 0.0908	– 0.0680	– **0.753**
	(0.0558)	**(0.152)**	(0.0610)	**(0.106)**	**(0.167)**	(0.0855)	(0.0775)	(0.0586)	**(0.932)**
Observations	5755	**2144**	6027	**1872**	**1006**	2763	4130	5445	**1452**
Effective F-statistic	37.85	**5.761**	35.08	**8.987**	**0.875**	13.21	24.51	32.32	**0.477**
WHO-5 Well-Being Index:									
WfH (dummy:1=yes)	0.672*	– **0.102**	0.524	**0.710**	– **1.177**	1.316**	0.259	0.530	**3.204**
	(0.389)	**(0.974)**	(0.422)	**(0.688)**	**(2.085)**	(0.658)	(0.492)	(0.415)	**(4.563)**
Observations	5718	**2132**	5982	**1868**	**1003**	2754	4093	5412	**1442**
Effective F-statistic	37.58	**6.451**	35.92	**8.451**	**0.773**	13.22	25.00	32.61	**0.476**

This table shows the estimated coefficients of 2SLS second stage regressions of Eq. 1 by subgroups for all dependent variables but *No. of sick days*. We refrain from testing heterogeneity for this outcome given the relatively small sample size and the non-significance of the effect of interest, cf. Table 8. Control variables are included as indicated in the respective equations. Standard errors reported in parentheses are clustered at the individual level for all regressions. The Effective F-statistic refers to Olea and Pflueger [50]

* $$p<0.10$$, ** $$p<0.05$$, *** $$p<0.01$$

## Data Availability

The data used in this paper, as described in Mackeben, Jan; Ruf, Kevin; Wolter, Stefanie; Grunau, Philipp; Graf, Tobias; Grießemer, Stephan; Kaimer, Steffen; Köhler, Markus; Lehnert, Claudia; Oertel, Martina; Seysen, Christian (2021): “Linked Personnel Panel (LPP) survey data linked to administrative data of the IAB (LPP-ADIAB) - Version 7519 v1”. Research Data Centre of the Federal Employment Agency (BA) at the Institute for Employment Research (IAB). 10.5164/IAB.LPP-ADIAB7519.de.en.v1, can be accessed via on-site use at the Research Data Centre (FDZ) of the German Federal Employment Agency (BA) at the Institute for Employment Research (IAB) and subsequently remote data access.

## Footnotes

1 These trends are similar to those exploiting other data sources, such as Eurostat (https://ec.europa.eu/eurostat/databrowser/view/lfsa_ehomp/default/table,, accessed on August 10th, 2023). However, a direct comparison is not possible due to the different sample designs and conditions of WfH frequency.

2 Sect. “Literature” reviews corresponding relevant studies.

3 Cf. https://www.bbc.com/news/technology-16314901 accessed August 10th, 2023, for the case of Volkswagen.

4 There are numerous factors potentially impacting individual health and well-being during a pandemic. For instance, Adams-Prassl et al. [17] show that government-imposed lockdowns are associated with lower well-being for women, even beyond what can be explained by childcare responsibilities.

5 Nevertheless, we perform a robustness check where we purge our regression sample from those who only spend few hours per week WfH ($<7$ hours/week). The results we discuss in the relevant section below are confirmed, with even more pronounced (significant) coefficients.

6 Unfortunately, information for the calculation of the individual number of days being sick is just available for the two last waves of the LPP resulting in a relatively smaller sample for this specification.

7 Detailed descriptive statistics on those variables can be found in Table 4 in the appendix.

8 Both pieces of information are taken from linking the LPP dataset with the Integrated Employment Biographies (IEB) dataset from the IAB.

9 This information has been exploited by linking the LPP to the IAB’s Establishment History Panel (BHP).

10 See Paulus and Matthes [46] for more information on the KldB identifier.

11 This phenomenon has also been encountered by other studies investigating the effects of WfH. For example, although exploiting a sample where individuals are observed for up to 15 years, Arntz et al. [25] are only able to exploit a within-variation in terms of taking up WfH by a magnitude of approx. 3%.

12 Other studies (e.g., Alipour et al. [48], Gottlieb et al. [49]) exploit occupation-specific or even task-based information on the feasibility to work from home to instrument actual (individual-level) usage. We employ similar measures as IVs in a robustness check, but find that the Effective F-statistics (Olea and Pflueger [50]) turn out too small, implying weak instruments.

13 For more information on the ATUS please see https://www.bls.gov/tus/, accessed August 10th, 2023.

14 Moreover, the significant difference in the share of the activity of *meetings by telephone or internet calls* can partly be attributed to its first component, which does not affect screen time.

15 Results are available upon request.

16 Given that the LPP surveys the individual number of sick days retrospectively, i.e., *No. of sick days* constitutes a flow variable, we substitute the outcome variable for the number of sick days in *t* with (i) its forward value, i.e., the individual value at $t+1$ as well as (ii) the mean of both. However, the results remain qualitatively the same, yielding a negative insignificant point estimate of WfH.

17 These effects are more pronounced when purging the sample from those who only spend few hours per week WfH ($<7$ hours/week). We take this as further support of the proposed underlying mechanism. However, this does not imply a linear relationship between health and the extent of WfH. Properly testing this would require more recent data from the pandemic where (much) more employees spent (much) larger amounts of time WfH, compared to our sample period when the majority of those regularly WfH worked from home for up to 8 h a week.

18 Unfortunately, due to the small sample sizes, our estimations do not always fulfill the above-mentioned instrument relevance condition. For ease of understanding, the 2SLS estimates in Table 9 that miss this relevance condition are bold.

19 We define commuters as persons whose establishment is located in a different municipality than his or her residence, excluding cases of larger cities for which our commuting identifier is not able to capture commuting behavior, since those cities, for instance Berlin, are coded and considered municipalities and most of the commuting in those cities is within the city borders.

## References

[1] Frodermann, C., Grunau, P., Haepp, T., Mackeben, J., Ruf, K., Steffes, S., Wanger, S.: Online-Befragung von Beschäftigten: Wie Corona den Arbeitsalltag verändert hat IAB-Kurzbericht 13/2020. IAB Nuremberg (2020)

[2] Hans-Böckler-Stiftung. Corona und Arbeitszeit: Lücke zwischen den Geschlechtern bleibt - Frauen erhalten seltener Aufstockung bei Kurzarbeit . [11.02.2021]https://www.boeckler.de/de/pressemitteilungen-2675-corona-und-arbeitszeit-lucke-zwischen-den-geschlechtern-bleibt-29563.htm (2020)

[3] Hans-Böckler-Stiftung. 2021. Deutlicher Anstieg: 24 Prozent der Erwerbstätigen arbeiten aktuell vorwiegend oder ausschließlich im Homeoffice . [17.02.2021]https://www.boeckler.de/de/pressemitteilungen-2675-deutlicher-anstieg-30681.htm

[4] Frodermann, C., Grunau, P., Haas, G.C., Müller, D.: Homeoffice in Zeiten von Corona: Nutzung, Hindernisse und Zukunftswünsche IAB-Kurzbericht 05/2021. IAB Nuremberg (2021)

[5] Welz, C., Wolf, F.: Telework in the European Union . Eurofund (2010)

[6] Mateyka, P., Rapino, M., Landivar, L.: Home-Based Workers in the United States: 2010 current population reports. US Census (2012)

[7] Vilhelmson B, Thulin E (2016). Who and where are the flexible workers? Exploring the current diffusion of telework in Sweden. N. Technol. Work. Employ..

[8] Vazquez, E., Winkler, H.: How is the Internet Changing Labor Market Arrangements?: Evidence from Telecommunications Reforms in Europe Policy Research Working Paper 7976. World Bank (2017)

[9] Autor D (2001). Wiring the labor market. J. Econ. Perspect..

[10] Singh, R., Orazem, P., Song, M.: Broadband Access, Telecommuting and the Urban-Rural Digital Divide Working Papers 18214. Iowa State University, Department of Economics. 10.22004/ag.econ.18214 (2006)

[11] Possenriede D, Hassink W, Plantenga J (2016). Does temporal and locational flexibility of work increase the supply of working hours? Evidence from the Netherlands. IZA J Labor Policy.

[12] Bloom N, Liang J, Roberts J, Ying Z (2015). Does working from home work? Evidence from a Chinese experiment. Q. J. Econ..

[13] Chu D, Akl E, Duda S, Solo K, Yaacoub S, Schünemann H, Schünemann H (2020). Physical distancing, face masks, and eye protection to prevent person-to-person transmission of SARS-CoV-2 and COVID-19: a systematic review and meta-analysis. The Lancet.

[14] Flaxman S, Mishra S, Gandy A, Unwin J, Mellan A, Bhatt TS (2020). Estimating the effects of non-pharmaceutical interventions on COVID-19 in Europe. Nature.

[15] Weber, E.: Which Measures Flattened the Curve in Germany? Covid economics 24. CEPR (2020)

[16] Kosfeld R, Mitze T, Rode J, Wälde K (2021). The covid-19 containment effects of public health measures: a spatial difference-in-differences approach. J. Reg. Sci..

[17] Adams-Prassl A, Boneva T, Golin M, Rauh C (2022). The impact of the coronavirus lockdown on mental health: evidence from the United States. Econ Policy.

[18] Hotz, V., Johansson, P., Karimi, A. (2018) Parenthood, Family Friendly Workplaces, and the Gender Gaps in Early Work Careers Working Paper 24173. National Bureau Econ Res. 10.3386/w24173

[19] van Ommeren J, Guterrez-i-Puigarnau E (2011). Are workers with a long commute less productive? An empirical analysis of absenteeism. Reg. Sci. Urban Econ..

[20] Chandola T, Booker C, Kumari M, Benzeval M (2019). Are flexible work arrangements associated with lower levels of chronic stress-related biomarkers? A study of 6025 employees in the UK household longitudinal study. Sociology.

[21] Li L, Wang S (2022). Do work-family initiatives improve employee mental health? Longitudinal evidence from a nationally representative cohort. J. Affect. Disord..

[22] Gariety B, Shaffer S (2007). Wage differentals associated with working at home. Mon. Labor Rev..

[23] Glass J, Noonan M (2016). Telecommuting and earnings trajectories among american women and men 1989–2008. Soc. Forces.

[24] Pigini C, Staffolani S (2019). Teleworkers in Italy: who are they? Do they make more?. Int. J. Manpow..

[25] Arntz M, Ben Yahmed S, Berlingieri F (2022). Working from home, hours worked and wages: heterogeneity by gender and parenthood. Labour Econ..

[26] Pabilonia S, Vernon V (2022). Telework, wages, and time use in the United States. Rev. Econ. Household.

[27] Rupietta, K., Beckmann, M.: Working from Home. Schmalenbach Business Review **70**(1), 25–55 (2018). 10.1007/s41464-017-0043-x

[28] Golden T, Veiga J (2005). The impact of extent of telecommuting on job satisfaction: resolving inconsistent findings. J. Manag..

[29] Troup C, Rose J (2012). Working from home: do formal or informal telework arrangements provide better work-family outcomes?. Commun Work Family.

[30] Possenriede, D., Plantenga, J.: Temporal and locational flexibility of work, working-time fit, and job satisfaction IZA discussion papers. Institute of Labor Economics (IZA) (2014)

[31] Kröll C, Nüesch S (2017). The effects of flexible work practices on employee attitudes: evidence from a large-scale panel study in Germany. Int J Hum Res Manag.

[32] Hansen K (2017). Home office—salutary action on combining work and family?. Die Unternehmung.

[33] Dutcher G (2012). The effects of telecommuting on productivity: an experimental examination. the role of dull and creative tasks. J Econ Behav Org.

[34] Nordberg M, Røed K (2009). Economic incentives, business cycles, and long-term sickness absence. Ind Relations J Econ Soci.

[35] Askildsen J, Bratberg E, Nilsen O (2005). Unemployment, labor force composition and sickness absence: a panel data study. Health Econ..

[36] Markussen S, Røed K, Røgeberg O, Gaure S (2011). The anatomy of absenteeism. J. Health Econ..

[37] Johansson P, Palme M (2002). Assessing the effect of public policy on worker absenteeism. J. Hum. Resour..

[38] Johansson P, Palme M (2005). Moral hazard and sickness insurance. J. Public Econ..

[39] Ziebarth N, Karlsson M (2010). A natural experiment on sick pay cuts, sickness absence, and labor costs. J. Public Econ..

[40] Gray, H.: CEP Discussion Papers 0529. Centre for Economic Performance, LSE (2002)

[41] Dex S, Smith C, Winter S (2001). Effects of family-friendly policies on business performance WP 22/2001.

[42] Heywood J, Miller L (2015). Schedule flexibility, family friendly policies and absence. Manch. Sch..

[43] Dionne G, Dostie B (2007). New evidence on the determinants of absenteeism using linked employer-employee data. ILR Rev..

[44] Possenriede, D., Hassink, W., Plantenga, J.: Does temporal and locational flexibility of work reduce absenteeism? Working Papers 14-09. Utrecht School of Economics (2014)

[45] Bech P, Olsen L, Kjoller M, Rasmussen N (2003). Measuring well-being rather than the absence of distress symptoms: a comparison of the SF-36 mental health subscale and the who-five well-being scale. Int. J. Methods Psychiatr. Res..

[46] Paulus, W., Matthes, B.: The German Classification of Occupations 2010 - Structure, Coding and Conversion Table FDZ-Methodenreport 08/2022. IAB Nuremberg (2013)

[47] Caliendo M, Mahlstedt R. Mitnik (2017). Unobservable, but unimportant? The relevance of usually unobserved variables for the evaluation of labor market policies. Labour Econ..

[48] Alipour JV, Fadinger H, Schymik J (2021). My home is my castle—the benefits of working from home during a pandemic crisis. J. Public Econ..

[49] Gottlieb C, Grobovšek J, Poschke M, Saltiel F (2021). Working from home in developing countries. Eur. Econ. Rev..

[50] Olea J, Pflueger C (2013). A robust test for weak instruments. J Bus Econ Stat.

[51] Bubonya M, Cobb-Clark D, Wooden M (2017). Mental health and productivity at work: does what you do matter?. Labour Econ..

